# Devolved health system capacity in the provision of care for sick newborns and young infants in four counties serving vulnerable populations in Kenya

**DOI:** 10.1371/journal.pgph.0000183

**Published:** 2022-10-20

**Authors:** Jesse Gitaka, Samuel Mbugua, Peter Mwaura, Daniel Gatungu, David Githanga, Charity Ndwiga, Timothy Abuya, Kezia K’Oduol, Wilson Liambila, Fred Were

**Affiliations:** 1 Directorate of Research and Innovation, Mount Kenya University, Thika, Kenya; 2 Kenya Paediatric Research Consortium, Nairobi, Kenya; 3 Population Council Kenya, Nairobi, Kenya; 4 Living Goods, Nairobi, Kenya; University of Alberta, CANADA

## Abstract

Possible severe bacterial infections (PSBI) is one of the three leading causes of newborn and young infant mortality globally that can be prevented by timely diagnosis and treatment using suitable antibiotics. High impact interventions such as use of out-patient injectable gentamicin and dispersible Amoxicillin with community-based follow up have been shown to reduce mortality in clinical trials. The objective of this study was to assess the health systems’ preparedness and organizational gaps that may impact execution in providing care for newborns and sick young infants. This formative research study was embedded within a three-year implementation research project in 4 Counties in Kenya. The indicators were based on facility audits for existing capacity to care for newborns and young infants as well as County organizational capacity assessment. The organizational capacity assessment domains were derived from the World Health Organization’s Health Systems Building blocks for health service delivery. The scores were computed by adding average scores in each domain and calculated against the total possible scores to generate a percentage outcome. Statistical analyses were descriptive with adjustment for clustering of data. Overall, the Counties have inadequate organizational capacity for management of sick young infants with Organizational Capacity Index scores of between 61–64%. Among the domains, the highest score was in Health Management Information System and service delivery. The lowest scores were in monitoring and evaluation (M&E). Counties scored relatively low scores in human resources for health and health products and commodities with one scoring poorly for both areas while the rest scored average performance. The four counties revealed varying levels of organizational capacity deficit to effectively manage sick young infants. The key underlying issues for the below par performance include poor coordination, low funding, inadequate supportive supervision, and M&E to enable data utilisation for quality improvement. It was evident that newborn and young infant health services suffer from inadequate infrastructure, equipment, staffing, and coordination. As Kenya, continuously rolls out the guidelines on management of sick young infants, there is need to focus attention to these challenges to enhance sustainable adoption and reduction of young infant morbidity and mortality.

## Background

It is estimated that 103 million neonates died between 1990 and 2017 [1]. 2.5 million neonates and 4 million infants died in 2017 and 2018 respectively, with 89% of the burden borne by low- and middle-income countries [1, 2]. 35% of all neonatal deaths in 2017 were related to preterm deaths, 24% to intrapartum events such as birth asphyxia, 14% due to sepsis and meningitis and 11% were associated with congenital anomalies [3]. These conditions contribute to 202 million disability-adjusted life years [4]. The neonatal mortality rate in Kenya was 22 deaths per 1000 live births [5]. Severe bacterial infection is one of the three leading causes of newborn and young infant deaths globally contributing to 20% of these deaths [6]. Reducing this burden requires strategies that result in timely case identification and initiation of suitable antibiotic treatment [7, 8]. This is challenging in places where most of the population does not have access to hospital-based care. Strategies to increase access to adequate services in low-income, high-burden settings are necessary as timely detection and appropriate case management can save hundreds of thousands of newborn and young infant lives [8]. Factoring current health system capacity for maternal, newborn and young infant care, it is estimated that two-thirds of countries in sub-Saharan Africa are at risk of missing the sustainable development goals on reducing neonatal mortality [1].

The WHO building blocks provide a common, structured discourse on health systems challenges and is recognized by policy makers and scholars globally [9, 10]. Health care systems in low- and middle-income countries continue to grapple with challenges in infrastructural and health resources capacity to tackle the burden that is newborn and young infants’ morbidity and mortality [11, 12]. A collaborative, partnership approach to strengthening health systems, improved health governance, building managerial capacity and engagement of private and public sectors remains an essential solution towards achieving effective coverage and improved equity across the care continuum [13]. Improvements in quality of care of neonatal health are linked to improved supervision while governance strengthening has positive implications for better resource utilization and user satisfaction [14]. Using a health systems approach, identifying barriers, and strengthening health systems functions using quality improvement teams improves participation and care to mothers, newborns, and young infants [15].

Over the past 3 decades there has been big efforts to upscale effective interventions with huge impact including the 2015 WHO guidelines on management of possible serious bacterial infection (PSBI) in young infants where referral is not possible [16]. Nonetheless, in real world conditions, evidence-based interventions are hardly implemented with absolute fidelity primarily because of existing capacity gaps [17]. Thus, there is need to understand capacity in health care facilities offering newborn and young infant care and identify resource and organizational gaps that may impact execution of interventions.

The Constitution of Kenya 2010 shifted the governance of service delivery and led to a devolved system with the establishment of 47 county governments and one national government [18]. The governments’ discreteness is determined by the Fourth Schedule of the Constitution, which assigned different functions to the two levels of government [19]. Article 186 assigns health service functions to the county governments [19]. The National Government continues to provide leadership and policy direction while the counties provide health service functions [20]. The study was conducted in 4 Counties in Kenya. The objective of this study was to assess the health systems’ preparedness in providing care for sick young infants in four counties. We also evaluated the implications of the health systems domains status on service delivery for newborns and young infants.

## Materials and methods

### Study design and context

This was a quantitative formative health systems study embedded in a larger three-year implementation research (IR) that sought to demonstrate the adoption of PSBI guidelines. The IR activities were implemented in 4 purposively selected Counties: County C represented a coastal urban sub-population of informal settlement representing socio-economic vulnerability compounded with complex social and physical access challenges. County D represented the coastal region with mixed rural/urban settings with geographical and cultural vulnerabilities. County B represented Western Kenya, a rural agrarian population, with physical access and cultural vulnerabilities. County A reflected a nomadic pastoralist lifestyle representing social, economic, cultural, and geographical vulnerabilities. Two sub-counties were selected in each county in consultation with the County Health Management Teams (CHMTs). The Health System Building Blocks assessed were Service Delivery Systems (Health service organization); Leadership and Governance (health service management); Health Workforce (Human Resources for the provision of Health Services); Health Financing (Resources for service provision); Health Products and Technologies (essential medicines, medical supplies, vaccines, health technologies, and public health commodities required in provision of services); Health Information (Systems for generation, analysis, dissemination, and utilization of health-related information); Health Infrastructure (physical infrastructure, equipment, transport, and Information Communication Technology needed.

#### Study participants

Key informants included key County and sub county management staff such as directors of health, chief officers of health or their designee, key program managers, heads of various departments and focal points for various service areas whose roles aligns to the health system domains. 4 CHMTs and 8 Sub County Health Management Teams (SCHMT) with 8–12 members were interviewed.

### Data collection

The assessment adopted a participatory, quantitative and consultative approach where the county teams reflected on the health system building blocks. In each domain (Table 1), three dimensions were examined: Status: if a given element exists, such as a policy or legislation; Quality: if the element conforms to established quality norms and Application: the extent to which the element is executed. Predetermined scores were designed to display in easy-to-interpret dashboards and spider plots [21]. Data collection activities were implemented between May and July 2018.

**Table 1:** Summary of domains assessed.

Domains	Focus of Questions
**Health Financing & Policy**	Policy formulation- development and adoption of National policies,
	Plans: county integrated development plans (CIDP), medium term expenditure framework (MTEF), Sector plans, Annual development plans (ADP), Annual work plans (AWP), Sector working group
	Budgeting: Circular, County Budget Review and Outlook Paper, Quarterly implementation reports, Audit reports, County Fiscal Strategy Paper, Program based budget, Revised estimates
	Budget execution and reporting: Procurement plan, cash flow forecast, financial reports, non-financial reports, Approval process (controller of budget), Timely reporting, quality of reports (content and structure), Use of integrated financial management information system (IFMIS)
**Human Resource Capacity**	Human Resource Policy/Plan/ County capacity to staff the county/sub-county/health facilities as per national Human Resource for Health (HRH) norms, guidelines and standards/ Employment database
	County capacity to strengthen staff performance management and supervision of existing workforce
	Ability to attract, recruit and retain health workers/Training and capacity development programme
**Leadership and Governance**	Leadership and Governance (political/leadership commitment, leadership capacity to meaningfully engage Program-based Budgeting (P&B) process
	CMHT structures & functionality /Partnerships and stakeholder coordination
**Health products and Technologies**	Governance and Oversight /Commodity Security/ Logistics Management
**Service Delivery**	Reproductive, Maternal, Newborn, Child and Adolescent Health (RMNCAH) services provided/ Service Charters /Supportive Supervision process and system
	Quality Improvement processes/ Referral system /Community facility Linkages and strategy
**Health Infrastructure**	Health Infrastructure requirements (includes medical equipment, emergency response system, buildings, technologies)
	Strategy: Health infrastructure policies and standards as per Ministry of Health (MoH) /Infrastructure Development & Execution /Budgetary allocation, Procurement plans, leasing arrangements (Public private partnership (PPP) & innovations)
**Health Information System**	Routine Data collection strategy/ Essential tool and equipment for data management (e.g. collection, transfer, storage, analysis) /Guidelines to document procedures for collecting, recording, collating and reporting routine program data
**Monitoring and Evaluation (M&E)**	M&E unit/focal person /An inventory/database for program evaluation /Forums for dissemination and discussion of evaluation findings

### Data analysis

#### Organizational Capacity Index

The Organizational Capacity Index (OCI) was computed quantitatively by adding average scores in each domain and calculating them against the total possible scores to generate a percentage outcome. For ease of interpretation, the overall OCI score was green if the counties scored 70%, amber for scores between 50–69% and red below 50%. The health policy is organized around the Health System Building Blocks [8] including **Service Delivery Systems** (health service organization); **Leadership and Governance** (health service management); **Health Workforce** (human Resources for the provision of Health Services); **Health Financing** (resources for service provision); **Health Products and Technologies** (essential medicines, medical supplies, vaccines, health technologies, and public health commodities required in provision of services); **Health Information** (systems for generation, analysis, dissemination, and utilization of health-related information); **Health Infrastructure** (physical infrastructure, equipment, transport, and Information Communication Technology needed).

*Ethics review and consent to participate*. This study was approved by the AMREF Ethics and Scientific Review Committee (as ESRC P430/2018) and the Population Council’s Institutional Review Board (as Protocol 838). Written informed consent was obtained from each participant before conducting an interview.

## Results

### Organizational capacity assessment index

Overall, the Counties have inadequate organizational capacity for management of sick young infants (SYIs) with PSBI. All counties scored amber cumulatively: County B and A scoring 61% while County C and D scored 64%. For each domain, the highest score was in Health Management Information Systems (HMIS) with an average score of 70% across counties and service delivery domain where County A, C and D scored a green, but B scored amber of 65%.

The lowest scores were in M&E where County B and County A scored red while the rest scored amber. Counties scored relatively low scores in human resources for health and health products and commodities with County A scoring red for both areas while the rest scored amber. The rest of the domains scored amber of between 51–65% (Table 2).

**Table 2:** Scores by health systems domains.

Organizational Capacity Index	County A	County B	County C	County D
Health Policy and Financing	69	65	69	67
Leadership and Governance	54	54	54	61
Human Resources for Health	54	49	58	61
Health Products and Technologies	61	42	56	62
Service delivery	67	71	74	71
Infrastructure	65	51	53	53
Health Management Information System	70	72	80	70
Monitoring and Evaluation	40	49	51	54
**Key**	Status	Score	Action needed	
< = 50	Red	Poor	Extensive support	
51–69	Amber	Average	Medium level support	
> = 70	Green	Good	Minimal support	

The leadership and governance structures demonstrated opportunities that may facilitate provision of PSBI services in the Counties. These include i) existence of important linkages between the County health Management Teams and the Sub County HMTs which enable coordination of activities across the 2 levels, ii) varying functionality of county level sector stakeholder’s coordination platform especially in counties with strong partners in child health services. However, there were critical gaps that will need to be filled for effective implementation of the PSBI guidelines. These included i) weak inter-sectoral coordination at sub county levels in Counties C and D and ii) CHMTs lack the resources and capacity to mentor, facilitate and build capacity of sector players weakening their functionality and iii) reduced focus by partners on newborn and young infant care, with skewed attention to maternal health.

#### Leadership and governance

Good leadership and governance play a vital role in the creation and pursuit of a shared vision for the health system. Key gaps are discussed below with priority areas that need to be acted upon for improving care provision in SYI;

- *Lack of a clear organogram outlining roles and responsibilities of the CHMT*. Most counties do not have a clear organogram. As a result, relative roles and responsibilities are unknown. In some cases, an organogram existed but it’s not updated or used for lower staff cadres.
- *Lack of structured training of health leadership at all levels*. There is no structured training of health leadership teams including orientation for new CHMT and SCHMT members. There also lacks a framework that works with the County Public Service Board for clear contractual arrangements for the CHMT.
- *Sup-optimal Functionality of CHMTs and SCHMTs*. Their functionality was sub-optimal with limited performance management and review structures. Additionally, the CHMTs lack the resources and capacity to mentor, facilitate and build capacity of sector players. There was no evidence of a capacity-building plan to strengthen the CHMT and ensure effective sector oversight, coordination, supervision, and monitoring. This is likely to affect management for SYI as capacity to oversee quality of service in primary health care facilities may be limited as well as the continuity of care from lower to higher level facilities.

*Weak public-private partnership structures in place and coordination*. This was characterized with unclear communication strategies. There is no clear county level structure, or a health department specific structure to manage private sector or other implementing partners. In general, counties do not have relevant personnel however for any new partner supporting the county health system a memorandum of understanding agreement is normally signed between the partner and county. Others do not have a framework by which donors can plug in their support frameworks so oftentimes the County is beholden to the support provided through donors. These findings may contribute to many partners implementing Maternal health, with limited focus given to SYI.

### Monitoring and evaluation

M&E assesses the performance of health projects, institutions, and programmes in relation to what was planned and the achievement of outputs, outcomes and impacts. The goal of the M&E system is to help improve health system performance and therefore lead to the achievement of the desired results. The M&E function is not well established at the county level with key gaps including:

- *Lack of M&E policies*, *frameworks and organizational structure*. In almost all counties, the County Health Departments do not have specific strategic policies, frameworks or organizational structures to lead and operationalize M&E functions. There are often no M&E units or focal persons and where a team exists, the unit is inactive and does not easily meet due to other responsibilities or it exists as part of the CHMT. In general, there are no links between the strategic plan and M&E plans since it there are no tracking systems for health system performance or tools to track M&E indicators.
- *Limited capacity to translate data into policy and practice*. Counties lack inventories for all relevant evaluations or research conducted in the counties. Where these exist, they are programme specific and donor funded. In general, there is no capacity in translating the data and research into policy and practice. Additionally, counties do not have effective forums for dissemination and discussion of evaluation findings, except at the CHMT level, nor do they have a clear way of developing information products to key stakeholders. Major capacity gaps in operational research and program evaluation systems exist, including lack of capacity to produce and disseminate credible and quality information to support evidence-based planning and decision making, ensure that feedback is provided throughout the health system to improve performance, accountability, service delivery, program implementation and practice, motivate demand and use of data or information at various levels of the health system and promote knowledge exchange and learning among stakeholders within the health system and sector.

### Performance in the domains

Different patterns of health systems capacity emerge demonstrating unique interplay within the domains. Health policy and planning domain scored amber for status and quality across sites with County D scoring a green for the application dimension. In terms of leadership and governance, all counties scored amber for status and quality except County A that scored a red for quality. All counties scored a red for the application dimension in this domain.

Regarding human resources for health all counties scored amber for the three dimensions except County D that scored a red for the application dimension. For health products and commodities, County B scored green in the status dimension, the rest scored amber for all dimensions except County A that scored red for the application dimension. Despite the challenges of infrastructure, there was an observation that service delivery area was still functional albeit with sub optimal quality of services. Good scores were reported for the status dimension with all counties scoring green and amber for quality. Counties A and D scored a green for the application dimension while the rest of domains scored amber.

HMIS scored green for status and quality for all counties but amber for application in Counties B, A and D, the latter being linked to the challenges of availability of tools for documentation, reporting process and data quality. The strength of this domain lies in the investment made over time even before devolution and the support given by various partners. M&E was the domain that scored low for all dimensions; County B scored red for three dimensions, the rest scored amber for status and quality.

**Fig 1:**
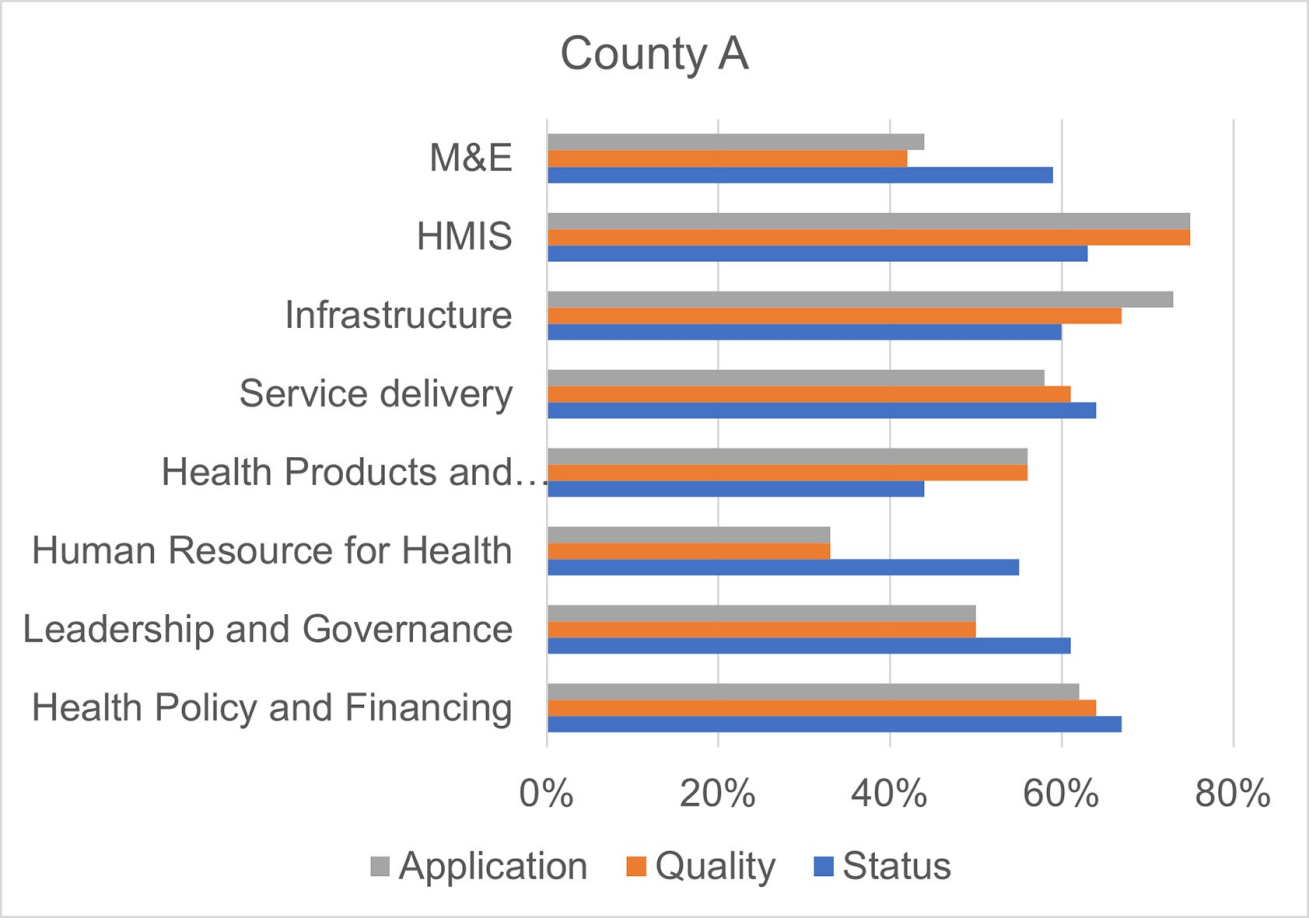
Scores by dimension of assessment, County A.

**Fig 2:**
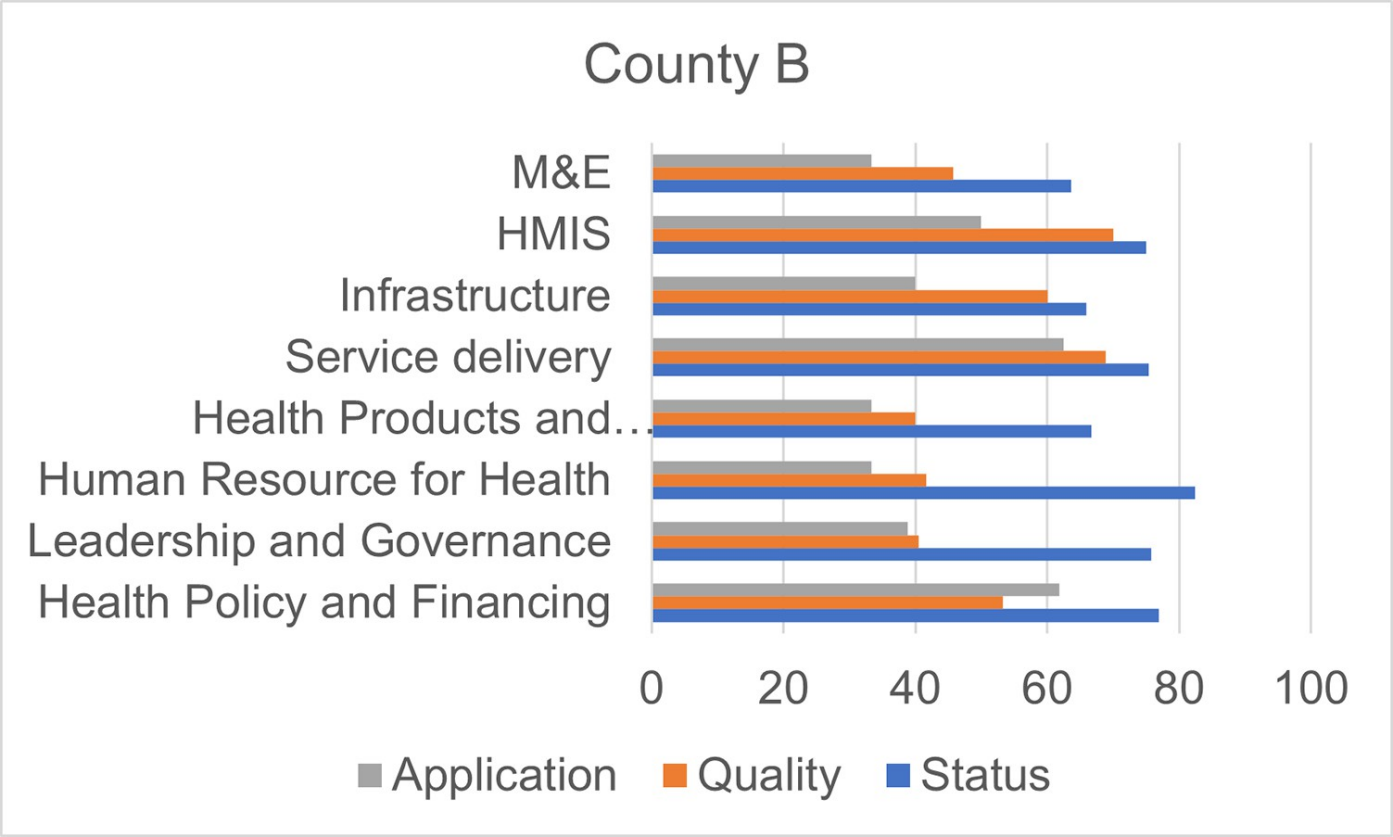
Scores by dimension of assessment, County B.

**Fig 3:**
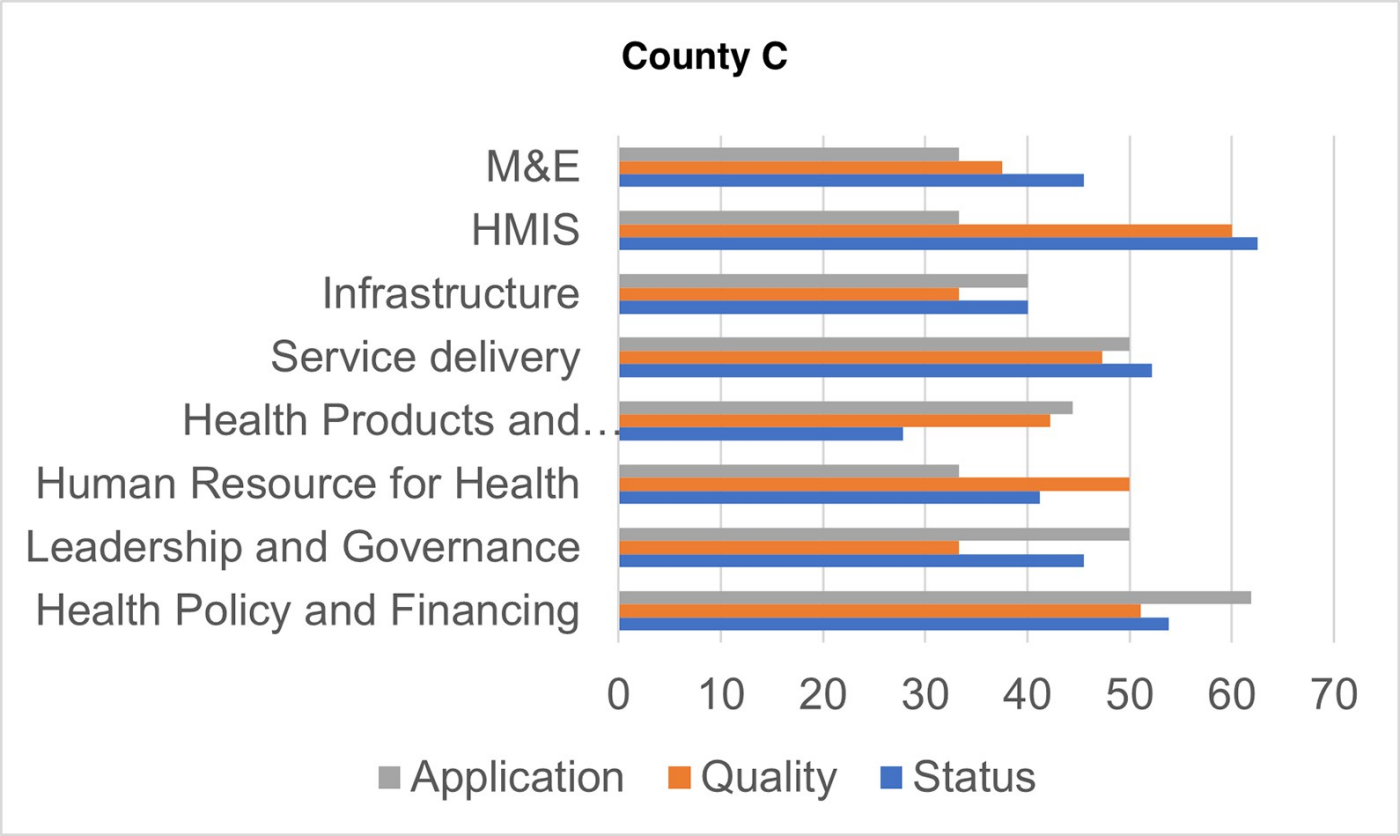
Scores by dimension of assessment, County C.

**Fig 4:**
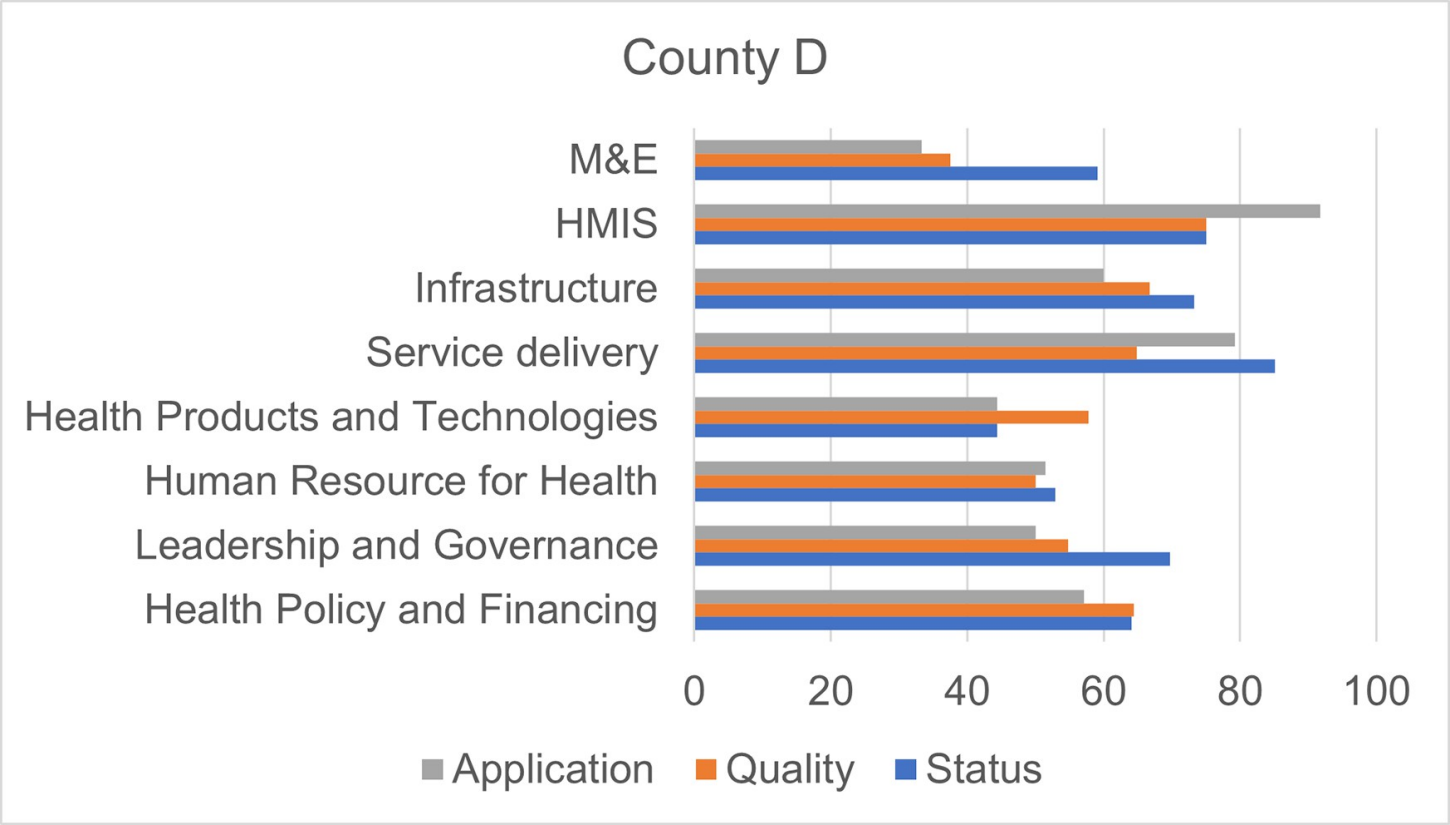
Scores by dimension of assessment, County D.

Figs 1–4 show the overall scores per domain in each county in terms of status, quality, and application.

### Leadership and governance

Our results show inadequate organization and coordination capacity of Integrated Management of Newborn and Childhood Illness (IMNCI) such as IMNCI focal person. In primary health facilities Maternal and Child Health unit (MCH) remain closed over the weekend which limits access to sources for SYI infants especially follow up for the newborn and young infant with SYI with PSBI on day 4 and day 8. Operational community units’ structures and community health volunteers (CHVs) trained on necessary identification of newborn and young infant danger signs, counselling and referral are also inadequate. There also lack champions for Maternal, Newborn and Child Health (MNCH) resulting in lack of prioritization of SYI management.

### Health products and technologies

Procurement of drugs especially antibiotics such as gentamycin and Amoxicillin at county level is inconsistent and inadequate due to poor disbursements of fund from the national governments to county government while on the other hand some county governments sometimes fail to honor payment to Kenya Medical Supplies Agency (KEMSA). CHMT and SCHMT were unaware of the PSBI guidelines but had IMNCI policy and strategies in place but very supportive of integration of the PSBI into existing PSBI service delivery points. WHO operational guidelines recommend effective coordination, planning and governance for IMNCI services that upon which PSBI will be anchored as part on integrated SYI services. There is need to enhance partner coordination, funding, planning and procurements of essential commodities and drugs to better leverage the available resources for MNCH especially for SYI with PSBI. Apart from County A, there are inadequate and inconsistency supply of essential medicines and commodities for management of SYIs across all the other counties. In counties A and B, there is weak forecasting, while counties D and B lacks quality assurance mechanisms for the commodities. County C is faced with poor distribution and utilization of antibiotics (prescription of drugs not recommended in IMNCI guidelines–more costly) for SYIs. Across all sites training for primary health care level providers on commodity/logistics management was inadequate.

### Human resource capacity for IMNCI services

There is inadequate number of staffs, and among them very few providers with capacity for IMNCI and PSBI management. Training for IMNCI has not been happening due to limited funding for IMNCI affecting Continuous Medical Education (CME) or mentorship thus capacity to assess, classify and treat SYIs especially at lower-level health care. Community Health Extension Workers (CHEWs) and CHVs are also not trained in community IMNCI. Human resource capacity necessary for management of SYI such clinical officers and nurses trained in IMNCI, pediatricians is in adequate across all sites. In County C and A, staff job descriptions, including for Maternal, Newborn Health (MNH) staff, have not been reviewed and rolled out.

### Health Infrastructure

Across all sites there is lack of effective supervision for maintenance of the MNH infrastructure as per norms and standards and poor transport infrastructure including grounded vehicles, lack of fuel and ill-equipped ambulances to support referral process including for SYIs. Specifically, to the new born, there are variations in capacity for infrastructure between sites. For example, in county C and D there is insufficient budgetary allocation for infrastructure development and maintenance while most health facilities in counties B and C lack space for newborn units and or there is crowding in newborn and young infant service areas where available. There is poor implementation of patient safety measures at health facilities in counties B and D.

### Service delivery for SYIs

As expected, many dispensaries operate Monday to Friday and hence services for SYI are unavailable over the weekends. Even though maternity services are available in many health centers during the weekend, there are no SYIs services. All counties have a referral policy and strategy adapted from the national governments. But there is inadequate operationalization of referral strategy in some counties. For example, county C lacks a designated referral focal person and is faced with a lack of ambulance /vehicle for referral. All the other counties have a referral focal person but experience vehicle breakdown and lack of fuel from time to time. There is a persistent financial resource gaps affecting planning and prioritization of service provision for SYIs exist in three of the four study counties (counties B, C and D). Identifying a focal person for IMNCI to coordinated Child health in the county and sub county may assist in planning services for SYIs. Service arrangements at referral/specialized is described as complex and deters caregivers to accept referral.

### Monitoring & evaluation and health information systems

Across all sites, District Health Information System (DHIS) is the data management platform. However, data on how SYIs are managed is not systematically available for monitoring progress. Also, indicators for SYIs are not overtly identifiable in existing HMIS data tools. At facility level, there is weak documentation in the Postnatal Care (PNC) registers including incomplete filling of data in some of the columns in the register due to lack of understanding or inadequate information on the type of data required to be filled. In some sites a few health facilities do not routinely report on a timely basis limiting use of routine data for decision making on MNH including SYIs service delivery. There is shortage of tools and registers across all the counties.

## Discussion

The four counties revealed varying levels of organizational capacity to effectively manage SYIs with PSBI. This has implications on the ability of these Counties to implement the 2015 WHO Guidelines on management of PSBI where referral is not feasible. To enhance the capacity and fill gaps on the particular domains, the following steps are recommended; increasing partner coordination to better leverage the available resources for MNCH especially for SYI; building provider capacity through mentorship or OJT or online virtual training as appropriate and encourage site-exchange visits for learning especially for sites that do not have providers trained in IMNCI and enhanced overall funding for newborn/young infant health. Health providers have shown motivation in the use of skills learnt in training initiatives to address work-related challenges as opined by Pfeiffer et al. [22] in a study on building health systems capacity to improve maternal and newborn care in Ghana. To facilitate the 4^th^ and 8^th^ day follow up and improve care seeking behaviors and decision support, there is need to conduct training/ or updates of CHVs on IMNCI, support supervision, and focus on their role in follow up for PSBI cases among other newborn and young infant illnesses. Continuous capacity building and support supervision are main-stream interventions aimed at improving health systems’ environment [23]. Sacks et al. [10] makes a case for the integration of community health in national health systems going beyond the building blocks and promoting prioritization and investment in community health. This indicates the pivotal importance of the community health system in service delivery.

Supply chain management is a critical component in ensuring availability of needed commodities such as dispersible Amoxicillin tablets, gentamicin and associated non-pharmaceutical goods such as syringes and needles. The accessibility, availability and effective utilization of essential medicines plays a critical role in delivery of quality health services [24]. To increase efficiency and reduce stock out, training providers on commodity/ logistics management and enhancing support supervision to support primary level health care providers on forecasting for IMNCI commodities among others will ensure timely/consistent procurement and distribution of antibiotics for SYI. Additionally, using the Laboratory Management Information System to improve reporting rate and decision making will enhance prompt care and utilization of services.

Newborn and young infant infrastructure was a key challenge for the counties with most facilities lacking dedicated spaces for newborn/young infant care and requisite equipment such as phototherapy machines, incubators, warmers and resuscitaires. An important contributor to this was low level funding for newborn and young infant services and inadequate support supervision to ensure maintenance and repairs were executed in time. This is reiterated by reiterated by Ayah et al. [24] and Barugahare & Lie. [25] that highlighted inadequate funding and poor priority setting as key challenges in MNH service delivery. Thus, Counties need to improve supervision for maintenance of MNH infrastructure as per norms and standards. This will ensure timely repairs and prevent further damage. Systematic supervision employing the use of clearly defined, quantifiable indicators has shown evidence of improved service delivery with modest budgetary implications [26]. Budgetary advocacy to increase allocations is necessary for optimal infrastructure and service delivery and ensure adherence to IMNCI guidelines implementation.

Ensuring availability of staff over the weekend in primary health facilities for PSBI follow up on days 4 and 8 advocacy to county, sub county and national governments for prioritization of IMNCI services and provision of requisite resources will fill financial and commodity gaps for IMNCI. Studies have identified staff shortage and retention related issues as important barriers to provision of quality care in MNH [27, 28].

On M&E, it was evident that data on how SYIs are managed is not systematically available for monitoring progress; Indicators for SYIs are not overtly identifiable in existing HMIS data tool; There was weak documentation in the PNC registers including incompletely filled data in some of the columns in the register due to lack of understanding or inadequate information on the type of data required to be filled; and reporting is not undertaken on a timely basis limiting routine data for decision making on MNH service delivery. These challenges were contributed mainly by shortage of tools and registers in the county and routine data quality checks are not optimally functionalized. To mitigate these, the Counties will need to adopt and use HMIS SYI registers for documenting of PSBI care and conduct support supervision to enhance routine SYIs data entry/recording and reporting.

### Strengths and limitations

Our study aimed at providing evidence on health systems’ preparedness and organizational gaps that exist potentially impacting the care provided to sick young infants. The findings had several strengths that included generalizability based on the selection criteria employed of a contextual mix of counties representative of Kenya’s health care system. Interrater reliability was ensured by use of a harmonized data quality abstraction guide while employing the structured questionnaire in data collection. Given the complexity of health systems especially at the micro level, the study had some limitations. The key informants were county and sub county health managers managing vast health systems domain hence depth of data provided was a challenge. The data provided may not be robust enough to infer causality in complex health system issues. Also, the overlapping roles of various departmental heads resulted in inconsistencies in data provided allowing us to map a wider range of evidence.

## Conclusion

The study revealed lack of preparedness in the four Counties on the care of SYI with PSBI looking at the health system domains as per the organizational capacity assessment. It was evident that newborn and young infant health services suffer from inadequate infrastructure, equipment, staffing, and coordination. Additionally, these are compounded by a weak supply chain, low supportive supervision, and inadequate utilization of M&E data to inform quality improvement. As Kenya, prepares to roll out the WHO 2015 PSBI guidelines in the coming months, there is need to focus attention on these challenges to enhance sustainable adoption and reduction of young infant morbidity and mortality.

## Supporting information

S1 File Health systems opportunities, gaps, and implications in management of sick young infants. (DOCX)
