# Supplementary material for: Devolved health system capacity in the provision of care for sick newborns and young infants in four counties serving vulnerable populations in Kenya
Source: PLOS Glob Public Health. 2022 Oct 20;2(10):e0000183. doi: 10.1371/journal.pgph.0000183 (PMC10022333; doi:10.1371/journal.pgph.0000183)
Supplement: S1 File — (DOCX) [file pgph.0000183.s001.docx]

| **Existing Opportunities within health system domains to improve management of SYI in primary health care facilities** | **Gaps** | **Implications for managing SYI with PSBI where referral is not feasible** |
| --- | --- | --- |
| **Governance and Leadership** | | |
| -Varying linkages between CHMT and SCMHT levels  -Level of priority for Maternal and Child Health varies by county largely dependent on existing partners and technical leads  -Limited functionality of county level health sector stakeholder coordination platform with counties that have strong partners having meaningful engagement | -Weak coordination mechanism for MNH services including young infant and newborn health.  -Partners and private sector participation in maternal and newborn health programs not well coordinated with skewed focus on maternal health  -Weak inter-sectoral coordination | -The weak linkages result in poor coordination on neonatal health service delivery.  -Lack of a comprehensive approach in MNH service provision negatively impacts the sustainability of public health interventions. |
| **Human Resources for Health** | | |
| -Existence of administrative officers with varying skill set to manage HRH  -Existence of focal person for newborn, child and adolescent health | -Staff job descriptions, including for MNH staff, have not been reviewed and rolled out (counties C and A)  -Very few specialized newborn care providers (pediatricians, clinicians) in the sub counties  -Limited periodic assessments of workforce needs to guide training and capacity development for IMNCI  -- Skills and knowledge gaps on IMNCI among health providers and CHVs with limited CME for primary health care providers | - Lack of essential frontline providers means a critical failure or strain in service delivery for SYIs with PSBI.  -Lack of specialized health workers translates to higher incidences of neonatal morbidity and mortality. |
| **Health Products and Technologies** | | |
| -Sub-county and health facility HPT committees exist (**counties B and C)**  -Average monthly consumption and stocks of essential drugs and commodities for facilities are monitored through LMIS (**counties B and A)**   - - Commodity Security TWG in place and meets quarterly.   Forecasting and quantification for essential commodities including MNH medicines is undertaken quarterly (county D) | -No proper storage facility for health products at both sub-county and facility levels  -Weak forecasting of commodities including essential medicines for SYIs at both sub-county and facility levels **(counties A and B)**  -Lack of/Weak quality assurance mechanism for newborn health commodities**(counties D and B respectively)**  **-** Inconsistency in procurement of antibiotics as well as poor distribution and utilization of antibiotics for SYIs (county C)  -Poor inventory management and inadequate skills for supply chain monitoring (**county C**)  **-** Weakness in governance of MNH products and commodities  -The LMIS is not used for decision making on health products and reporting rates are below 10%. (county D) | Weak forecasting of essential commodities results in stockouts of critical medicines in the management of SYIs with PSBI.  Frontline providers require periodic re-orientation and induction to PSBI and the quality assurance procedures around it through support supervision and quality improvement teams.  Inefficient commodities’ management translate to increase in the burden of disease on the health system. |
| **Health Infrastructure** | | |
| -County’s health infrastructure is functional with most facilities having basic medical equipment and other MNCH infrastructure needed to provide basic services  - | -Insufficient budgetary allocation for infrastructure development and maintenance**(counties C and D)**  -Lack of space for newborn units and crowding in newborn service areas**(counties B and C)**  -Poor implementation of patient safety measures at health facilities**(counties B and D)**  -Lack of effective supervision for maintenance of MNH infrastructure as per norms and standards  -Poor transport infrastructure including grounded vehicles, lack of fuel and ill-equipped ambulances to support referral process | Compromised, inefficient provision of care for SYIs.  -The quality of care provided is negatively affected to lack of funding for appropriate interventions. |
| **Service Delivery** | | |
| -Facilities provide maternal and newborn health services according to existing standards and guidelines  -Existence of community strategy with varying levels of functionality of community units  -Structures for service quality improvement existing but not optimally implemented | -Inconsistent supportive supervision for MNH services  -Newborn services in lower level facilities are not offered round the clock  -Inadequate operationalization of referral strategy  -inadequate support for community strategies to manage community level activities  -Poor implementation of IMNCI guidelines | Lack of support supervision means poor uptake of PSBI and IMNCI interventions  -Inadequate referral pathway, lack of support for community strategies negatively affects care seeking and timely care delivery for SYIs. |
| **Health Financing and Policy** | | |
| -Program-based budgeting utilized in MNH planning.  -Costed work plans with result framework included in annual work plans for MNH and other health services | -Inadequate financing of sub-county MNH and other healthcare activities  -Delays in procurement of health supplies, including MNH care commodities due to budget constraints and prioritization  -MNCH programs are partly incorporated in the County’s health sector plans but are largely donor-driven  Persistent financial resource gaps affecting planning and prioritization of service provision for SYIs | The implementation of PSBI guidelines and strengthening of IMNCI strategies is compromised due to inadequate financial resources and investment in MNH service provision.  -Donor support for critical services results in loss of sustainability if and when the funding is withdrawn. |
| **Monitoring and Evaluation and Health Management information system** | | |
| - Routine health management information system in place with tools to capture essential indicators for routine monitoring  --Monitoring and evaluation indicators are tracked through the DHIS and data summary tools  -Information generated from M&E data is disseminated at in-charges’ meetings and data review meetings in some counties (County B) | -Data on how SYIs are managed is not systematically available for monitoring progress  -Indicators for SYIs are not overtly identifiable in existing HMIS data tools  -Weak documentation in the PNC registers including incomplete filling of data in some of the columns in the register due to lack of understanding or inadequate information on the type of data required to be filled  - Reporting is not undertaken on a timely basis limiting routine data for decision making on MNH service delivery  -Shortage of tools and registers in the county  -Routine data quality checks not optimally functionalized | -develop and use HMIS SYI registers for documenting of PSBI care  -conduct support supervision to enhance routine SYIs data entry/recording and reporting |
